# Mechanosensitive channel MscL induces non-apoptotic cell death and its suppression of tumor growth by ultrasound

**DOI:** 10.3389/fchem.2023.1130563

**Published:** 2023-03-01

**Authors:** Xiaoxu Wen, Yingying Wang, Zhenya Zhu, Shuangshuang Guo, Junjie Qian, Jinjun Zhu, Zhenni Yang, Weibao Qiu, Guofeng Li, Li Huang, Mizu Jiang, Linhua Tan, Hairong Zheng, Qiang Shu, Yuezhou Li

**Affiliations:** ^1^ National Clinical Research Center for Child Health, Children’s Hospital, Zhejiang University School of Medicine, Hangzhou, China; ^2^ Department of Biophysics, Institute of Neuroscience, NHC and CAMS Key Laboratory of Medical Neurobiology, Zhejiang University School of Medicine, Hangzhou, China; ^3^ Department of Biophysics and Kidney Disease Center, The First Affiliated Hospital, Zhejiang University School of Medicine, Hangzhou, China; ^4^ Department of Biophysics, Department of Neurology of the Fourth Affiliated Hospital, Zhejiang University School of Medicine, Hangzhou, China; ^5^ Paul C. Lauterbur Research Center for Biomedical Imaging, Shenzhen Institute of Advanced Technology, Chinese Academy of Sciences, Shenzhen, China; ^6^ Shenzhen Key Laboratory of Ultrasound Imaging and Therapy, Shenzhen, China; ^7^ School of Biomedical Engineering, Guangdong Medical University, Songshan Lake Science and Technology Park, Dongguan, China

**Keywords:** MscL, mitochondrial permeability, low intensity focused ultrasound, cytoplasmic vacuolization, nanopore

## Abstract

Mechanosensitive channel of large conductance (MscL) is the most thoroughly studied mechanosensitive channel in prokaryotes. Owing to its small molecular weight, clear mechanical gating mechanism, and nanopore forming ability upon opening, accumulating studies are implemented in regulating cell function by activating mechanosensitive channel of large conductance in mammalian cells. This study aimed to investigate the potentials of mechanosensitive channel of large conductance as a nanomedicine and a mechano-inducer in non-small cell lung cancer (NSCLC) A549 cells from the view of molecular pathways and acoustics. The stable cytoplasmic vacuolization model about NSCLC A549 cells was established *via* the targeted expression of modified mechanosensitive channel of large conductance channels in different subcellular organelles. Subsequent morphological changes in cellular component and expression levels of cell death markers are analyzed by confocal imaging and western blots. The permeability of mitochondrial inner membrane (MIM) exhibited a vital role in cytoplasmic vacuolization formation. Furthermore, mechanosensitive channel of large conductance channel can be activated by low intensity focused ultrasound (LIFU) in A549 cells, and the suppression of A549 tumors *in vivo* was achieved by LIFU with sound pressure as low as 0.053 MPa. These findings provide insights into the mechanisms underlying non-apoptotic cell death, and validate the nanochannel-based non-invasive ultrasonic strategy for cancer therapy.

## 1 Introduction

Mechanisms underlying paraptosis, pyroptosis, necroptosis and ferroptosis are extensively defined, which provides new insights for the investigation of non-apoptotic cell death (Sperandio et al., 2010; Wang et al., 2017; Wang et al., 2019a; Fontana et al., 2020; Liu et al., 2020). Previous studies indicated that pore forming proteins (PFPs) such as BAX/BAK, mixed lineage kinase domain-like (MLKL) and gasdermins (GSDs) play a significant role in addressing tumor resistance *via* non-apoptotic cell death (Shi et al., 2015; Flores-Romero et al., 2020; Martens et al., 2021; Garciaz et al., 2022). Therefore, the pore-forming components for the purpose as triggered nanopores are extensively investigated in cell death.

MscL can be activated by enhanced membrane tension during cell swelling upon hypoosmotic challenge (Blount & Iscla, 2020). It opens a non-selective pore (∼30 Å in diameter), which ions and small molecules below 1 kDa can be easily passed through (Van den Bogaart et al., 2007). The nanopore may result in the rapid disruption of intracellular homeostasis. Heureaux team reported that MscL expressing MDA-MB-231 cells showed defective cell migration (Heureaux-Torres et al., 2018). A recent study showed that curcumin induced membrane permeabilization was caused by MscL channel (Wray et al., 2021). Heterologous expression of MscL in HepG2 cells was shown the similar functional consequence to that of PFPs, and the severity of cell death could be furtherly modulated by the opening probability, gating kinetics and the pore size of MscL (Wen et al., 2020). Thus, MscL presents the role of nanomedicine as an inducer of cytoplasmic vacuolization leading to non-apoptotic cell death.

Cytoplasmic vacuolization refers to the predominant vacuoles visible in the cytoplasm, which commonly derives from dilated endoplasmic reticulum (ER) or swelling mitochondria (MITO) in cancer cells treated with anticancer compounds (Yoon et al., 2014; Chen et al., 2018). Cytoplasmic vacuolization cell death can target cancer cells due to the abnormal proliferation and metabolism in cancer (Barlett et al., 2006). Owing to the extremely complex causes and involved signaling molecules, the mechanism underlying cytoplasmic vacuolization is poorly understood. A major challenge is to establish a reliable model with a stable cytoplasmic vacuolization phenotype. MscL could be an excellent candidate inducer. Utilizing a MscL-based model for cytoplasmic vacuolization cell death and clarifying the formation of vacuoles may help understand the mechanism of cytoplasmic vacuolization and elucidate its role in cell death.

Existing collection of MscL mutants extends the mechanosensor with diverse mechanosensitivities, and MscL channels have been modified to respond to several stimuli including small compounds, ultrasound, pH, and temperature (Doerner et al., 2012; Yang et al., 2018; Ye et al., 2018; Owada et al., 2019). Ultrasound activated MscL was shown the ability to regulate apoptosis of tumor cells (He et al., 2021). Compared to other mechanosensitive channels, MscL is directly gated by membrane tension and none of other proteins or ligands are needed for the activation in any membrane structure. It has reliable functional remodeling due to clear background research. It responds to ultrasound in the absence of microbubbles. Its small gene size makes the engineered expression simple. Activated MscL channel can be viewed as a nanopore for the transportation of small molecule drugs and cytosolic contents.

Sonogenetics based on MscL may achieve potential application for the non-invasive control of cell death, which indicates the correlative study of cancer and biomedical engineering. Here, we further extended the potential of MscL as a mechano-inducer of cytoplasmic vacuolization cell death from the view of molecular pathways and acoustics. We established a stable cytoplasmic vacuolization model in NSCLC A549 cells by the targeted expression of modified MscL channels in different subcellular organelles. The permeability of mitochondrial inner membrane-MIM was shown a vital role in cytoplasmic vacuolization. Underlying mechanism of cytoplasmic vacuolization cell death has crosstalk with other types of cell death by analyzing the subsequent morphological changes and expression levels of cell death markers. Furthermore, we achieved the suppression of A549 tumor *in vivo* by a relatively low intensity of focused ultrasound *via* MscL activation.

## 2 Materials and methods

### 2.1 Cell culture

A549 and human embryonic kidney 293FT cells were grown in Dulbecco’s modified Eagle’s medium supplemented with 10% fetal bovine serum (FBS, Gibco) in the presence of penicillin/streptomycin (100 U mL^-1^). Scepter3.0 handheld automated cell counter (Merck) was used for cell counting.

### 2.2 Plasmid constructs

*Escherichia coli (E. coli)* MscL cDNA was codon-optimized for mice expression. Construction of codon-optimized MscL cDNA into the plasmid was performed by seamless cloning. 6xHis-T2A-mCherry was added into the lentiviral vector to gain lenti6.3/V5-CMV-MscL-6xHis-T2A-mCherry using the ClonExpress II One Step Cloning Kit (Vazyme Biotech co., ltd). Similarly, mCherry was fused to the pmCherry-N1 vector to obtain N1-MscL-mCherry. Subcellular located sequences (KDEL for ER retention, SRL for peroxisomal targeting, COXVIII presequence for MIM targeting, membrane-targeting myristoylated sequence, Myr) were ligated into N1-MscL-mCherry vectors for expressing MscL in different subcellular localizations. All-in-one doxycycline inducible lentiviral system (tet-on) was constructed based on the pCW57-MCS1-P2A-MCS2 (Neo) vector, which was a gift from Adam Karpf (RRID: Addgene_89180). We introduced MscL-6xHis-T2A-mCherry to MCS1 site and replaced neo resistance with puromycin. All vectors were sequenced with both strands to confirm successful construction. COX8 presequence: MSVLTPLLLRGLTGSARRLPVPRAK, membrane-targeting myristoylated sequence: MGSSKSKPKDPSQRRRRPGSAAA.

### 2.3 Generation of stable cell lines expressing MscL

293 FT packaging cells were transfected with the lentivial vector plenti6.3/V5-MscL-6xHis-tag and lentiviral packaging system which is a mixture of pLP1, pLP2, and pLP/VSVG vectors using Lipofectamine3000 (Invitrogen), 48 h or 72 h later, the supernatant was collected and filtered (Millipore 0.45 µm). After being concentrated by BioVision’s PEG Virus Precipitation Kit (Catalog #K904-50, BioVision), the acquired virus was used for infection of A549 cells in the presence of polybrene (8 μg mL^−1^, SIGMA, H-9268). After antibiotic selection for three generations (Blasticidin-S, 6 μg mL^−1^, Solarbio, B9300), the percent of mCherry-expressed cells reached 90%, stable clones were obtained. For the doxycycline inducible lentiviral system, preparation of lentivirus and infection of A549 cells were performed as described above. Stable cell lines of A549 expressing tet-on-MscL were gained by puromycin (1 μg mL^−1^, Solarbio) screening, the expression of MscL was confirmed by mCherry fluorescence imaging and electrophysiology recording after doxycycline (1 μg mL^−1^, MB1088, Dalian Meilun) induction.

### 2.4 Electrophysiological recordings

The electrophysiological recordings were performed according to a previous report (Wen et al., 2020). In brief, MscL single channel currents were recorded from Axopatch 200B and Digidata 1440A system (Axon Instruments/Molecular Devices). For excised inside-out recordings, 145 KCl, 5 NaCl, 2 MgCl_2_, 1 EGTA, and 10 HEPES (in mM) were in the bath buffer. 145 NaCl, 5 KCl, 5 CaCl_2_, 10 HEPES, and 2 MgCl_2_ (in mM) were in the pipette buffer. All electrophysiological recordings were operated at room temperature. Negative pressure applied to the membrane patches were operated by the HSPS system (ALA Scientific).

### 2.5 Western blots

Whole cell extracts were harvested according to the instruction (BC3710, Solarbio). Extract concentration was measured by BCA kit (SK1070, Coolaber, Beijing) on microplate reader (M5, Molecular Devices). Cell extracts were separated on 4%–20% SurePAGE Bis-Tris gels (Genscript) and then transferred to PVDF membranes (Millipore). Membranes with proteins were incubated with primary antibodies to be tested at 4°C overnight, then were incubated with fluorescence or HRP conjugated secondary antibodies for 1 h at room temperature. Odyssey SA system was used to detect His tagged MscL, and chemiluminescence was examined by ECL reagent (SL1350, Coolaber, Beijing). The densitometry was determined using Bio-Rad ChemiDoc Touch imaging system (1708371, Bio-Rad). Primary antibodies are as follows: Caspase-3 (9664T, cell signaling technology-CST), Caspase-3 (9746T, CST), Caspase-9 (9502T, CST), Ubiquitin (#58395, CST), Cytochrome-C (#11940, CST), LC3A/B (#12741, CST), TLR4 (ab13556, abcam). Secondary antibodies are as follows: anti-rabbit (8889S, CST), anti-rabbit (ab6721, abcam).

### 2.6 MTT assay

MTT assay was used to measure relative cell viability according to a previous report (Wen et al., 2020). In brief, A549 cells were seeded on 96-well plates with 4 k per well. 10 μL MTT labeling reagent (final concentration is 0.5 mg mL^−1^) was added to each well after the treatment finished, 100 μL DMSO was added after incubation for 4 h. The 96-well plate was then allowed to incubate at 37°C for 10 min in a shaker. The absorbance for every well at 490 nm was acquired with M5 microplate reader (Molecular Devices). Heterologous expression actually causes cell toxicity. To better compare the cell viability influenced by different mutants, the transient expression of MITO-mTFP1 and G26C-mCherry was used to be control for Figures 2F, G, respectively.

### 2.7 Live cell imaging

Confocal imaging was performed in A549 cells with transient overexpression system induced by lipo-transfection. For each organelle, the controlling of variable was applied. That is, 150 k A549 cells were seeded on 35 mm cell culture dish, and the transfection was performed after 24 h. The transfection system was composed of opti-MEM (200 μL), lopo3000 (5 μL), P3000 (5 μL), plasmids (2 μg), and the incubation time was 8 min at 37°C. The concentration of each plasmid was around 1,000 ng/μL. We controlled the overall consistence in expression level as described above. KDEL, SRL, COXVIII, and Myristoylated coding sequence-linked mTFP1 were used to indicate the subcellular localization of the ER, PERO, MITO, and plasm membrane, respectively. Nuclear envelope localization protein mEmerald-laminB1 and Hoechst 33342 (Invitrogen) were used to stain cell nuclei. mEmerald-LaminB1 was a gift from Michael Davidson (RRID: Addgene_54140) (Day & Davidson, 2009). Cell images were captured on FluoView-3000 laser scanning confocal microscope and analyzed by FluoView-3000 software (Olympus).

### 2.8 Ca^2+^ fluorescence

Calcium probe of fluo4-AM (F14201, Invitrogen) was used to detect the changes in intracellular Ca^2+^. Cell cultures were incubated 30 min with fluo-4 AM in HBSS containing Ca^2+^ (1.26 mM) and Mg^2+^, then 3 mL HBSS per confocal dish were added after three times washes with HBSS. Real-time changes of intracellular calcium were acquired by FluoView-3000 laser scanning confocal microscope at an ex/em of 488/515 nm in the time-lapse mode. Obtained data were analyzed with Image J software.

### 2.9 Cell derived xenografts in mice

All animal procedures complied with the ARRIVE guidelines and were approved by the Animal Ethical and Welfare Committee of Zhejiang Chinese Medical University (Approval Number: IACUC-20220124-01). Athymic BALB/c nude male mice were provided by Shanghai SLAC Experimental Animal Co. Nude mice of 4–6 weeks old and weighing 16–18 g were used. A549 cells, I92GI96G-MscL, and tet-on-V23A-MscL stable cells (5.0 × 10^6^) were subcutaneously injected into the right back of nude mice to establish xenograft tumors. Six days after injection (the length or width of the tumor reached 5 mm), the tet-on-V23A-MscL mice group was administered doxycycline (2 mg mL^−1^) in drinking water and renewed every 2 days. Three days later, mice were sonicated for 10 min for 2 weeks. Tumors were collected and processed for immunohistochemistry. All mice were weighed every 4 days. The xenograft tumor size was measured every 4 days.

### 2.10 Ultrasound application

The device of low intensity focused ultrasound consisted mainly of controller and transducer. The controller was offered by Shenzhen Institute of Advanced Technology, Chinese Academy of Sciences. The point target focus (PTF) transducer (code: V314-SU) was purchased from Olympus Corporation. The sound pressure can be regulated by the changing the settings of amplitude percentages in the controller. Under the condition of keeping the constant setup of T1 (Pause Width, 500 µs), T2 (Pause Interval, 1 ms), T3 (Stimulus Duration, 1 s) and T4 (Stimulus Interval, 3 s), the sound pressure of 100% amplitude is 171 kPa (equal to Isppa:91.2 mW cm^−2^ and Ispta:15.2 mW cm^−2^, respectively).

### 2.11 Statistical analysis

Image J software was used for the analysis of confocal images. Three to five independent assays were obtained for analysis. Analysis of multiple treatments was performed by one-way ANOVA using GraphPad Prism version 9.4.1 for Windows (Serial Number: GPS-2527509-EIU4-69ED7). Significance was indicated by *p* < 0.05 (in detail: *p < 0.05, **p < 0.01, ***p < 0.001, ****p < 0.0001).

## 3 Results

### 3.1 MscL is functionally expressed in A549 cells

**FIGURE 1:**
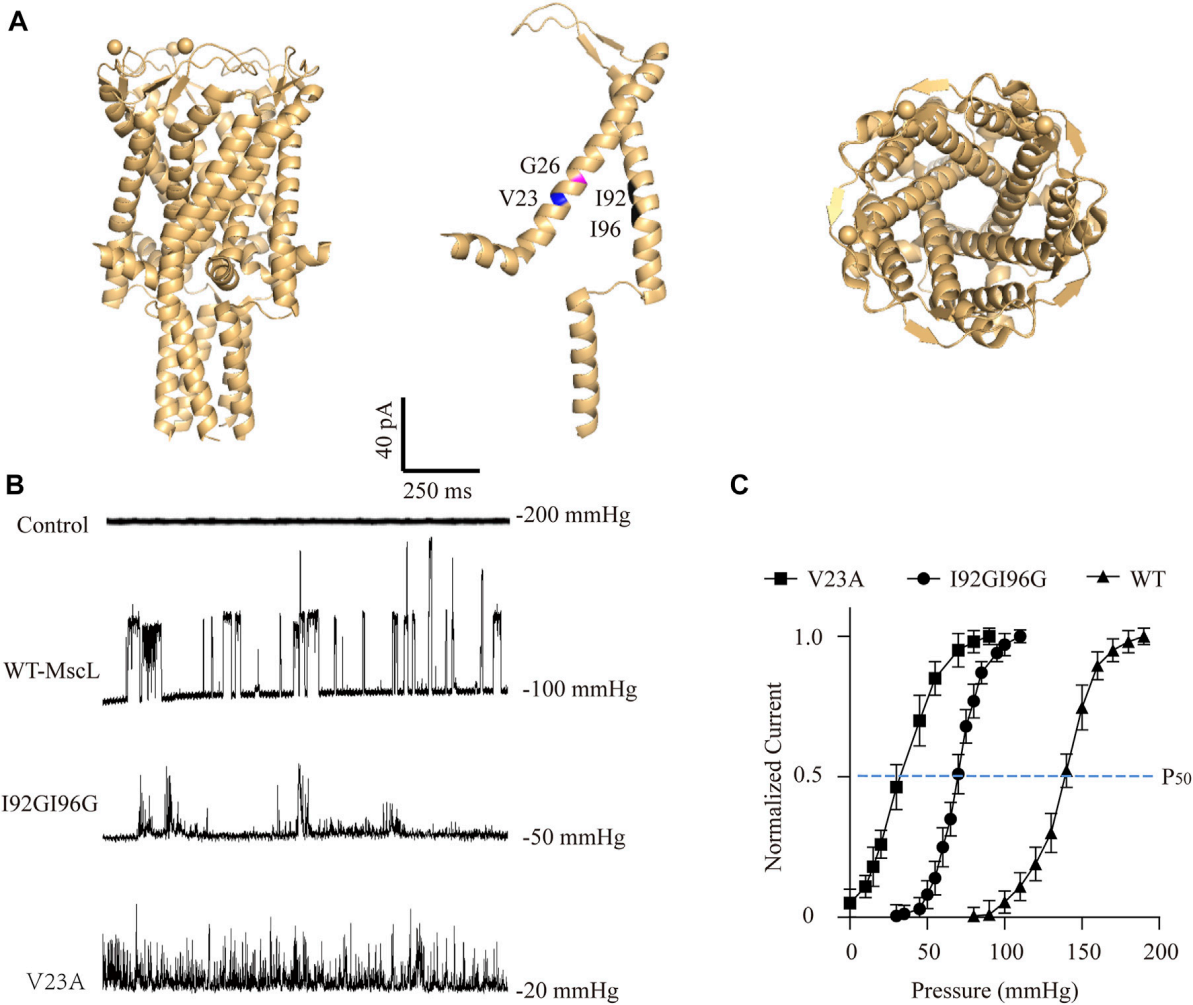
Electrophysiological analysis of the MscL channels expressed in A549 cells. **(A)** Illustration of the crystal structure of MscL channel (PDB 20AR). Amino acid sites-Val23 (blue), Gly26 (magenta), Ile92 and Ile96 (black) are highlighted. **(B)** Traces show single channel activity at the indicated holding pressure in Vhold = +40 mV. **(C)** Normalized current-pressure relationship of WT, I92GI96G and V23A MscL channels recorded from inside-out patches.

To investigate the potential cellular responses upon MscL gating, three MscLs with different mechanosensitivities were employed as graded force sensors. WT-MscL and known severe gain of function (GOF) mutation, V23A-MscL (replacing valine at site 23 with alanine), were expressed in A549 cells by lentiviral transfection (Li et al., 2004; Doerner et al., 2012). In addition, an engineered MscL mutant that both isoleucine at sites 92 and 96 were substituted with glycine (I92GI96G-MscL) were generated. I92 and I96 are critical residues located in the TM2 transmembrane α-helix (Figure 1A) and substitutions may lead to modified mechanosensitivity through TM1-TM2 interaction (Chang et al., 1998; Rees et al., 2000; Li et al., 2009).

As expected, negative pressure induced large single-channel currents about 21.43 ± 8.11 pA were recorded in WT-MscL-expressing A549 cells, while none of mechanosensitive current could be detected by increasing negative pressure up to 200 mmHg from control cells (Figure 1B). Different from the obvious full-opening states of WT-MscL, V23A, and I92GI96G exhibited flickery activities and higher mechanosensitivities of MscL channels. The gating threshold for WT-MscL in A549 cells was −99.17 ± 4.17 mmHg, and which was moderate sensitive for I92GI96G as 48.96 ± 11.78 mmHg. As a severe GOF mutant, V23A showed extreme mechanosensitivity that partial spontaneous gating could be observed even in the absence of negative pressure (Figure 1C). Consistently, the leftward shift of the dose−response curve revealed a pressure for half-maximal activation of the V23A and I92GI96G (P50, −35 ± 14 mmHg and −70 ± 17 mmHg, respectively) lower than that of the WT (P50 = −140 ± 11 mmHg). The electrophysiological analysis demonstrated functional expressions of MscL channels in A549 cells, and supported the intermediate mechanosensitivity of I92GI96G-MscL between that of WT and V23A.

### 3.2 Construction of subcellular-localized expression and doxycycline inducible system of MscL

**FIGURE 2:**
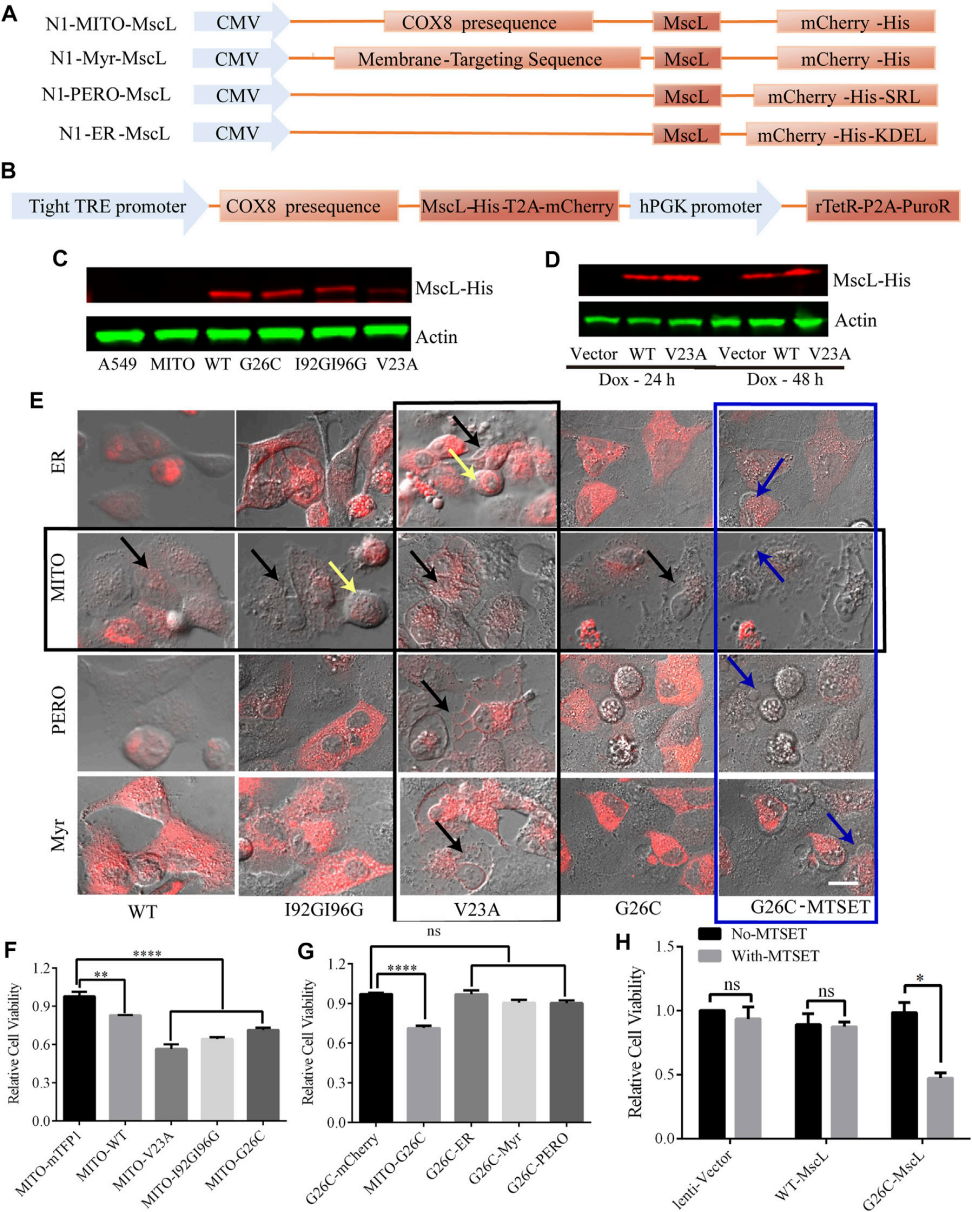
Subcellular expression and doxycycline-inducible expression of MscL channels in A549 cells. **(A)** Graphical representation of the vector constructs of expressing MscL in different subcellular localizations. **(B)** Graphical representation of the vector constructs of expressing MscL in doxycycline (dox) inducible lentiviral system. **(C)** and **(D)** Western blot detection of MscL-His of transiently expressing MITO-located MscL and doxycycline-induced stably expressing MITO-located MscL in A549 cells. **(E)** Morphology of control and MscL-mCherry expressing cells when MscL channels were located in subcellular sites of ER, MITO, PERO, and cell membrane, yellow, black, and blue arrows indicate cell swelling, cytoplasmic vacuolization and membrane blebbing, respectively. **(F)** and **(G)** Summary of cell viability of A549 cells transfected with different MITO-located GOF-MscL channels and different subcellular localizations of G26C-MscL by MTT assay (mean ± SEM, **p < 0.01, ****p < 0.0001, ns: no significance). **(H)** MTT assay detected the effect of MTSET on lentivirus stable cell lines of A549, (mean ± SEM, *p < 0.05, ns: no significance). Scale bar: 10 μm.

Intracellular membrane system is important to cytoplasmic vacuolization, especially the vacuoles were believed to originate from the expanded ER due to the imbalance in cell homeostasis (Ohoka et al., 2017). To determine the impact of permeability changes in different subcellular membranes on cytoplasmic vacuolization formation, we constructed MscL-expressing vectors targeting ER, MITO, and peroxisome (PERO), and plasma membrane (Myr) (Figure 2A). Since the overexpression of GOF mutation of MscL such as V23A could cause cytoplasmic vacuolization associated cell death (Figure 2F). High cell lethality introduced problems for the production of stable A549 cell line expressing V23A-MscL that may be used for cell-derived xenografts in an animal model. As an alternative, a doxycycline-induced tet-on system was constructed to achieve a controllable expression of V23A-MscL under temporal and spatial requirements (Figure 2B). Co-localizations of expressed MscL with targeted organelle were examined by the merge of the fluorescences from organelle-mTFP1 and MscL-mCherry (Supplementary Figure S1). Meanwhile, the overexpression of MscLs in subcellular localization and the doxycycline inducible system were confirmed by western blots (Figures 2C, D).

### 3.3 Activating MscL caused cytoplasmic vacuolization and decreased cell viability in A549 cells

The MscL open pore could initialize the leakage of cytosol and the subsequent abnormal morphologies such as cytoplasmic vacuolization and related cell swelling and membrane blebbing were detected in MscL-expressing A549 cells when targeted in different subcellular organelles. First, the overexpression of V23A-MscL can stably cause cytoplasmic vacuolization regardless of subcellular localization (**vertical black square,** Figure 2E). Next, chemically modified MscL mutant G26C (replacing glycine at site 26 with cystein) was involved as positive control of MscL opening (Bartlett and Iscla, 2006). G26C itself showed the similar gating property to WT, but binding with thiosulfonate reagent [2-(trimethylammonium) ethyl] methane thiosulfonate bromide (MTSET) allowed the G26C channel to be triggered and further locked in opening state. With MTSET treatment, expression of G26C in any subcellular localization could induce membrane blebbing, which associated with severe phenotype (**blue square,** Figure 2E). Interestingly, for the WT, I92GI9G and G26C without treatment, abnormal morphologies can only be observed when they are expressed in MIM of A549 cells (**horizontal black square,** Figure 2E), indicating the important role of permeability of MIM. Moreover, the morphological changes of MITO-located MscL-expressing A549 cells presented mutant dependent phenotype: partial cytoplasmic vacuolization (MITO-WT and MITO-G26C without MTSET), both cytoplasmic vacuolization and cell swelling (MITO-I92GI96G), prominent cytoplasmic vacuolization (V23A) and prominent membrane blebbing (G26C with MESET). These data suggested that increased permeability of MIM was tightly associated with cytoplasmic vacuolization. In addition, the progressive trend in morphological changes may be accompanied by the degree of MscL opening including open possibility, duration, and pore size.

To assess the impact of increasing permeability of MIM on cell proliferation, we measured the viability of MscL expressing A549 cells *via* MTT assay. Compared with vector, expression of MscL in MIM could cause decreased cell viability, the severity of which was gradually exacerbated with the degree of MscL opening (Figure 2F). Meanwhile, expressing G26C-MscL in MIM induced significant inhibition of cell viability comparing with other subcellular localizations (Figure 2G). Compared with lentivirus-vector and WT-MscL-expressing A549 cells, decreased viability of G26C-MscL-expressing cells was seen after MTSET treatment (Figure 2H). Overall, increased permeability of MIM induced by the nanopore of MscL resulted in cytoplasmic vacuolization and decreased cell viability.

### 3.4 MscL-induced cytoplasmic vacuolization related with deformation in the subcellular composition

**FIGURE 3:**
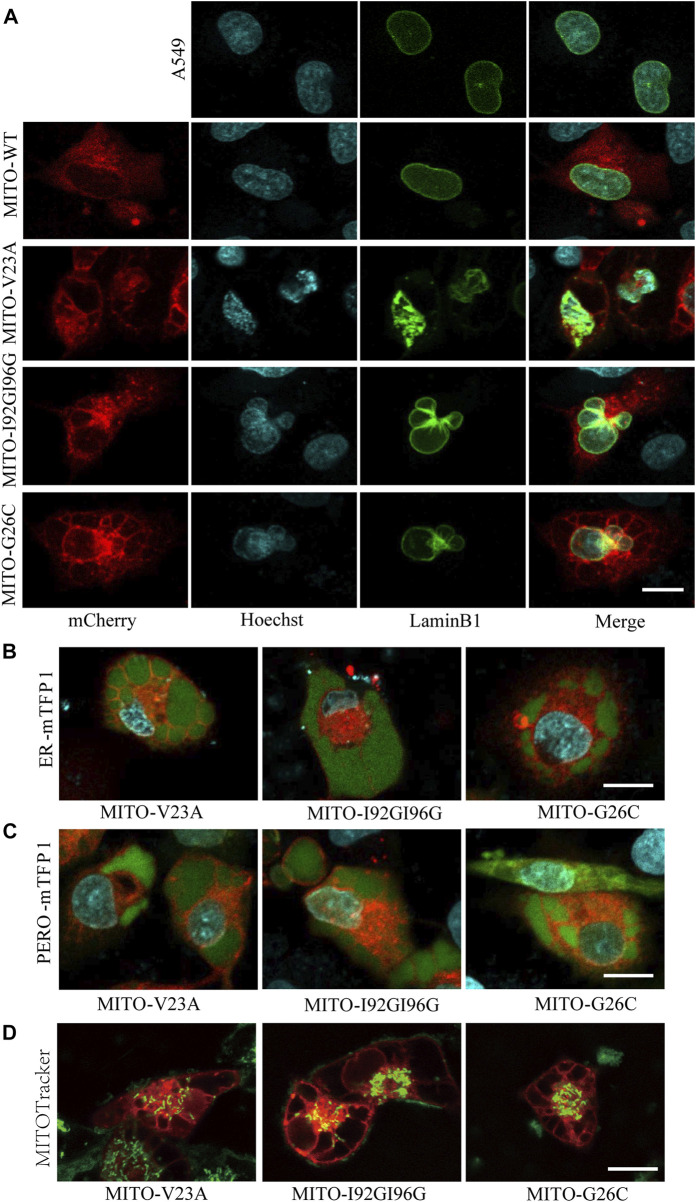
Mitochondrial expression of MscL induced cytoplasmic vacuolization in A549 cells. **(A)** Confocal imaging of nucleus morphology of MscL-expressing A549 cells visualized with overexpression of LaminB1 (Ne) protein and Hoechst (DNA) staining. **(B)** and **(C)** Morphology of mTFP1-marked ER and PERO of A549 cells with cytoplasmic vacualization. **(D)** MITOTracker indictes the mitochondrial morphology of vacuolated A549 cells. Scale bars: 10 µm.

Next, we assessed the changes in subcellular component caused by MscL opeing in A549 cells. Impairments of nucleus morphology are a common phenotype in multiple types of cell death. Further analysis of confocal imaging showed that vacuolated cells have extremely irregular nuclear morphology (Figure 3A), suggested that nuclei deformation associated with different periods of cytoplasmic vacuolization cell death or morphological changes.

Our previous work demonstrated that cytoplasmic vacuolization was associated with ER (Wen et al., 2020). Herein, we detected the morphological changes in ER and PERO by co-expression of MscL with subcellular localization targeted vectors. Confocal images revealed that the vacuoles were filled by the swelling ER and PERO, demonstrating that the vacuoles in cytoplasmic vacuolization were originated from both ER and PERO (Figures 3B, 3C). Meanwhile, disrupted morphology and reduced numbers of mitochondria in vacuolated cells were detected (Figure 3D). These results supported the disrupted cellular structure from impaired homeostasis induced by increasing permeability of the plasma membrane due to the nanopores formed by MscL opening.

### 3.5 Molecular mechanisms underlying cytoplasmic vacuolization cell death

Above results indicated the critical role of MIM in cytoplasmic vacuolization. Protein and RNA synthesis inhibition experiments suggested the involvement of new protein and RNA in the further process of cytoplasmic vacuolization induced by MscL (Supplementary Figure S2A). Live cell imaging indicated that the change from cell shrinkage to membrane blebbing following cytoplasmic vacuolization shared similarity with apoptosis and necrosis. We then used mitochondria expressed MscL (tet-on-MITO-MscL) as a controllable model to induce stable cytoplasmic vacuolization in A549 cells. Total proteins of lentivirus stable cells and tet-on-MITO-MscL-A549 at 24 h and 48 h after doxycycline induction were collected for Western blot.

**FIGURE 4:**
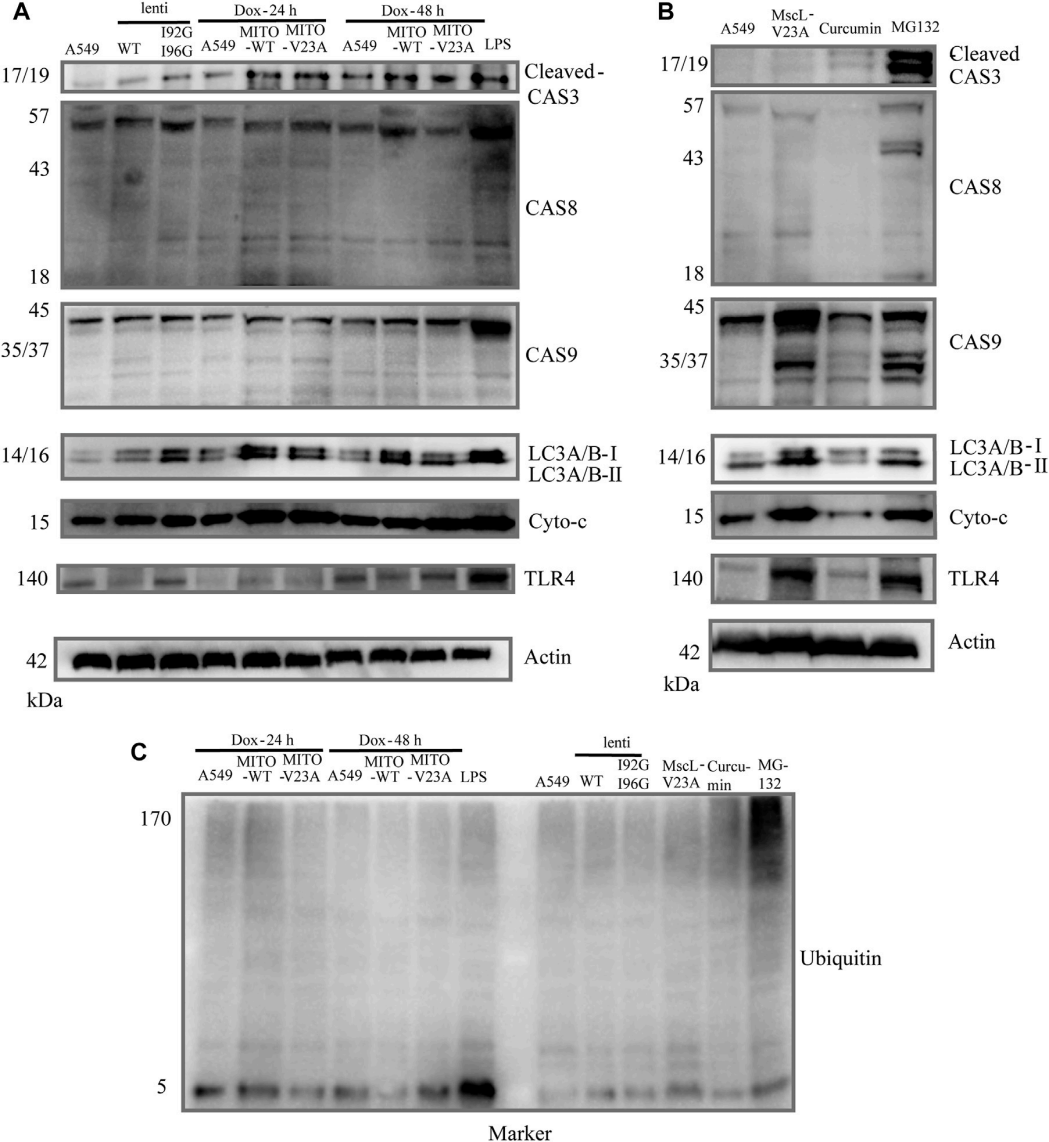
Molecular mechanism of cytoplasmic vacuolization. **(A)** Cell lysates from doxycycline-induced MITO-MscL expressing A549 cells were analyzed by immunoblotting with the annotated antibodies. **(B)** Cell lysates from different types of cytoplasmic vacuolization were analyzed by immunoblotting with the annotated antibodies. **(C)** Western blot analysis of ubiquitin. LPS (1 μg/mL, 12 h), Curcumin (40 μM, 12 h), MG-132 (25 μM, 16 h).

To clarify the relationship of apoptosis and autophagy to cytoplasmic vacuolization, the expression of caspase-3, caspase-8, caspase-9 and light chain 3 A/B (LC3A/B) were tested in A549 cells. No obvious active caspase-8 or active caspase-9 were measured in vacuolated A549 cells (**upper panel,** Figure 4A). Moreover, active cleaved caspase-3 was implicated in the formation of cytoplasmic vacuolization. However, z-VAD-FMK which is a pan-caspase inhibitor cannot suppress the cytoplasmic vacuolization (Supplementary Figure S2A). Consistent with previous studies (Kar et al., 2009; Zheng et al., 2016), increased endogenous LC3A/B-II protein was detected in vacuolated cells, demonstrating the involvement of LC3A/B-II protein in cytoplasmic vacuolization other than autophagy (**middle panel,** Figure 4A). Confocal imaging showed membrane blebbing happening in 48 h after doxycycline induction treatment differed from the morphological changes in apoptosis and pyroptosis (Supplementary Figures S2B, C). In addition, the expression of cytochrome-c and toll like receptor (TLR4) were measured. Lipopolysaccharide (LPS) treatment was used to induce the overexpression of TLR4. TLR4 showed slight changes in doxycycline-treated A549 cells 48 h after addition, whereas no different changes between MITO-WT- and MITO-V23A-MscL-expressing cells (**lower panel,** Figure 4A). We supposed that was due to the disrupted intensity of plasma membrane along with cytoplasmic vacuolization. Doxycycline-induced-MscL-expressing vacuolated A549 cells showed an increase in cytochrome-c expression, especially at the induction of 24 h, which means the crosstalk with apoptosis at the early stage of cytoplasmic vacuolization. These results indicated that cytoplasmic vacuolization may need or activate cleaved caspase-3 but bypass initiator caspase members. For lentivirus stable A549 cells, increased LC3A/B and TLR4 were tested in I92GI96G expressing A549 cells, however, the influence on cells needs further investigation.

Moreover, to compare the signaling pathway molecules underlying different types of cytoplasmic vacuolization cell death, we established different models of cytoplasmic vacuolization caused by overexpression of V23A-MscL, MITO-V23A-MscL, curcumin, and MG132. Compared with cytoplasmic vacuolization caused by increased permeability of MIM due to the overexpression of MITO-V23A-MscL, the active caspase-8 and caspase-9 and increased endogenous LC3A/B-II protein were involved in V23A-MscL- and MG132-induced cytoplasmic vacuolization, whereas active caspase-3 was only involved in MG132 treatment (**upper panel,** Figure 4B). Compared with cytoplasmic vacuolization caused by MG-132 and overexpression MITO-V23A-MscL, no obvious increased endogenous LC3A/B-II protein was detected in cytoplasmic vacuolization caused by curcumin (**middle panel,** Figure 4B). For V23A-MscL-expressing A549 cells, disrupted intensity of plasma membrane is certain. Increase in cytochrome-c and obvious expression of TLR4 were tested in V23A-MscL-expressing and MG132 treated A549 cells, which may indicate the disrupted membrane after MG132 treatment (**lower panel,** Figure 4B). Comparing to the ubiquitinated proteins in MG132-induced cytoplasmic vacuolization, no ubiquitinated proteins were detected in MscL overexpression and curcumin treatment, demonstrating the absence of proteasomal dysfunction in vacuolated A549 cells induced by mitochondrial overexpression of MscL (Figure 4C).

In summary, the four types of cytoplasmic vacuolization of MITO-V23A overexpression, V23A-expression, MG-132 and curcumin showed no fully consistent signal pathways. Although similar patterns of cytoplasmic vacuolization were visible, the molecular mechanisms underlying cell death caused by the expression of MITO-located pure V23A-MscL and V23A-MscL were different. These treatments represent the different mechanisms underlying cytoplasmic vacuolization cell death caused by increased permeability in MIM or plasma membrane, and/or impairment in proteasome function. These results indicated that the formation and development of cytoplasmic vacuolization require signal pathways coupled with apoptosis, autophagy, mitochondrial function, and proteasomefunction. However, specific signaling molecules of cytoplasmic vacuolization need further study.

### 3.6 LIFU induced calcium influx in A549 cells by activating I92GI96G-MscL

**FIGURE 5:**
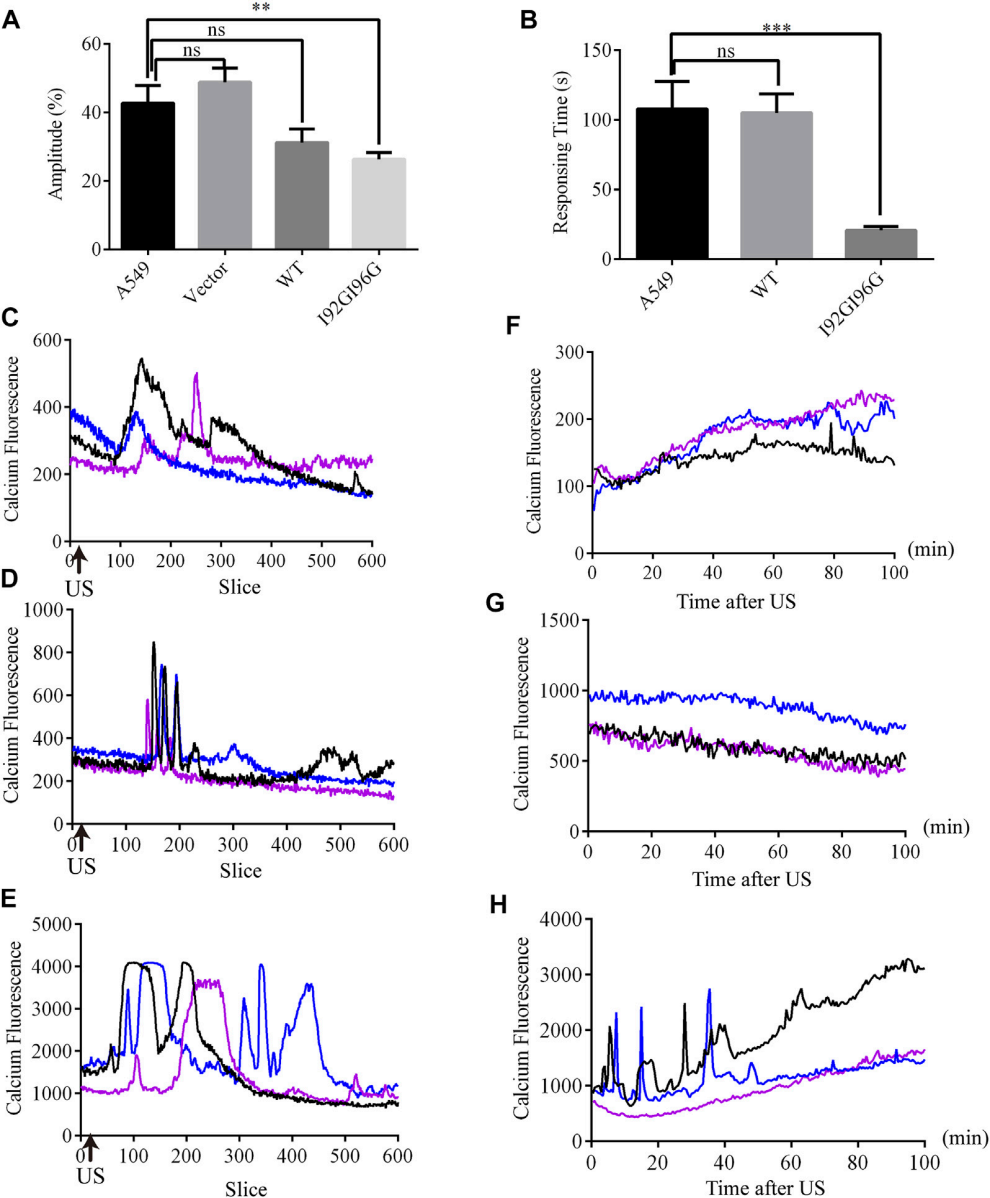
LIFU activates MscL channels in A549 cells. **(A)** The statistics of minimum sound pressure that caused fluo4 fluorescence change (mean ± SEM, **p < 0.01, ns: no significance). **(B)** Response time needed for A549 cells at the sound pressure of 0.053 MPa (equivalent to Isppa: 91.2 mW cm−^2^ Ispta: 15.2 mW cm−^2^) (mean ± SEM, ***p < 0.001). **(C–E)** Recordings of real-time calcium fluorescence when A549 **(C)**, WT-MscL. **(D)** or 192G196G-MscL, expressing A549 cells **(E)** were treated with ultrasound at the sound pressure of 0.053 MPa, arrows indicate the onset of ultrasound. **(F–H)** Recording of calcium fluorescence after treated 10 min with ultrasound at the sound pressure of 0.053 MPa in A549 **(F)**, WT-MscL **(G)** or I92GI96G-MscL expressing A549 cells **(H)**.

Although overexpression of MITO-V23A-MscL is capable of suppressing A549 cells proliferation, the adjustability of spontaneous MscL channels is limited. More MscL modifications and kinds of mechanical stimuli are expected. Electrophysiological recordings showed that I92G96G-MscL maybe an excellent candidate for further application due to its intermediate degree of open and mechanosensitivity. Moreover, ultrasound is becoming a popular type of mechanical stimulation in biophysics due to accurate spatial localization and non-invasiveness (He et al., 2021; Zhang et al., 2021). Herein, we expect to inhibit tumor cell growth with ultrasound by introducing MscL with as low sound pressure as possible in lentivirus constructed stable A549 cells. Calcium influxes were employed to indicate the activation of MscL. Compared with control A549 cells that occurred calcium influx at the average sound pressure of 0.073 MPa (43% Amplitude), WT-MscL and I92GI96G-MscL expressing cells underwent calcium influx in 0.053 MPa (31% Amplitude) and 0.044 MPa (26% Amplitude), respectively (Figure 5A). Herein, I92GI96G-MscL expressing A549 cells were activated after ultrasound stimulation at the average sound pressure of 0.044 MPa. The results indicated that A549 cells became more sensitive to ultrasound after expressing I92GI96G-MscL.

Moreover, WT-MscL expressing cells needed on average 105.12 ± 18.87 s to respond to ultrasound at the sound pressure of 0.053 MPa (31% Amplitude), and I92GI96G-MscL expressing cells only needed 20.72 ± 18.87 s (Figure 5B). Compared with control and WT-MscL-expressing A549 cells, I92GI96G-MscL expressing cells indicated short interval and higher response frequency and shorter duration to ultrasound at the sound pressure of 0.053 MPa (Figures 5C–E). Ten minutes after ultrasound, confocal microscopy tracking showed that calcium fluorescence of I92GI96G-MscL expressing A549 cells experienced sustainable elevated or calcium spark compared to the slight quenching in control cells (Figures 5F–H). As shown in Figures 5C–H, concentration of cytosolic calcium in I92GI96G-expressing A549 cells is higher than control and WT-MscL-expressing cells. The activating sound pressure for MscL was lower than previously reported in sonodynamic therapy, PIEZO1 and trek1 (Kubanek et al., 2016; Xu et al., 2021; Song et al., 2022). Here, we successfully increased the mechanosensitivity of A549 cells by introducing MscL channels and used a relatively low intensity of focused ultrasound to activate MscL channels.

### 3.7 LIFU inhibits A549 tumor growth by activating MscL

**FIGURE 6:**
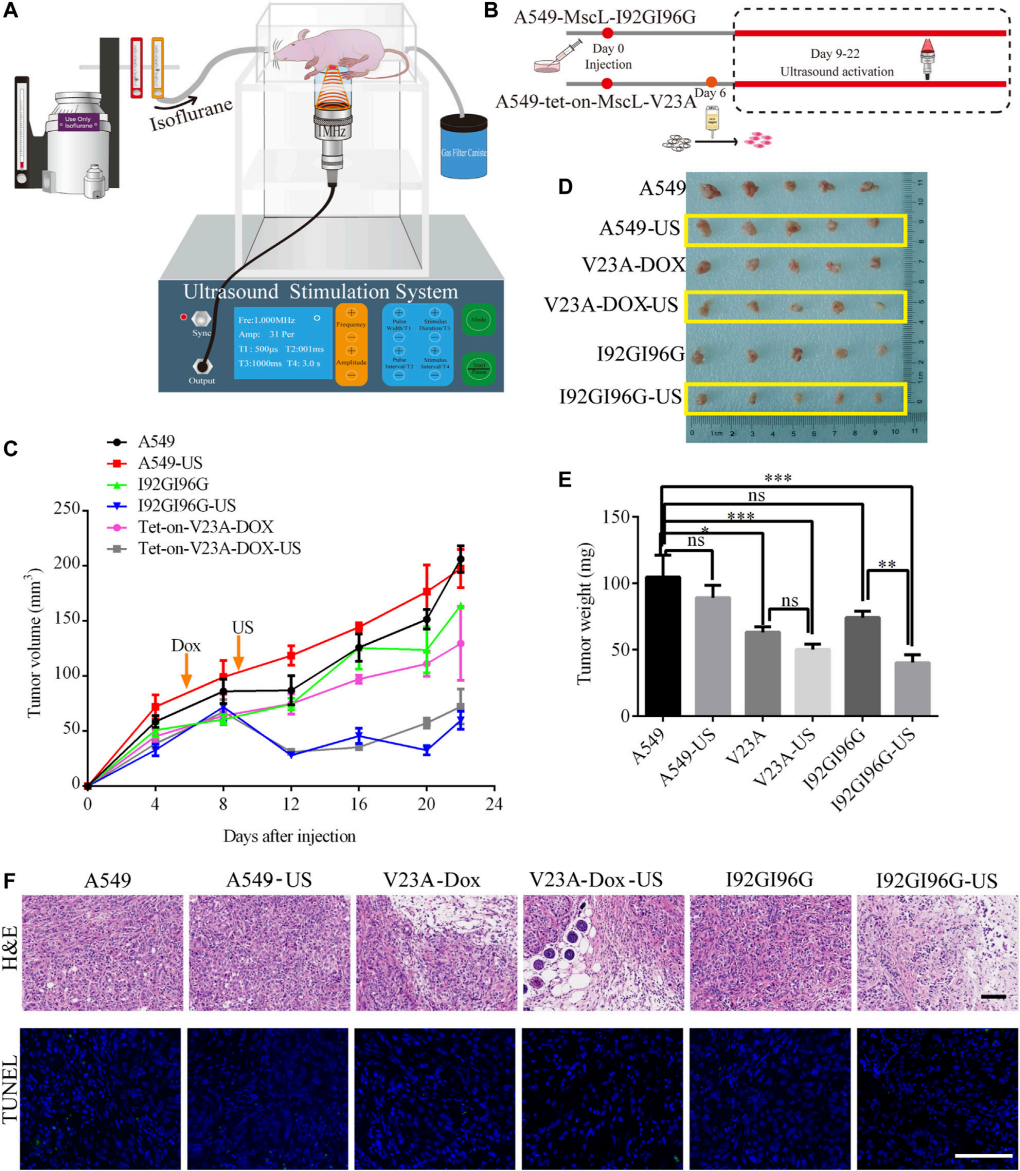
LIFU inhibits A549 tumor growth through activating MscL. **(A)** Schematic illustration of the experimental set-up for ultrasound BALB/c nude mice. **(B)** Schematic diagram of A549 xenograft in BALB/c nude mice. **(C)** Summary of tumor growth of different mouse groups (mean ± SEM, *n* = 5). **(D)** Mass of tumor samples after 22 days of transplantation. **(E)** Summary of tumor weight following sacrifice of mice (mean ± SEM, *n* = 5, *p < 0.05, ***p < 0.001, ns: no significance). **(F)** Representative areas of H&E and TUNEL staining of tumor tissue. Scale bars: 50 µm.

We next examined the suppression of LIFU on A549 tumors through MscL channel in BALB/c nude mouse model. A549 cells, I92GI96G-MscL or tet-on-V23A-MscL expressing A549 cells were subcutaneously injected into mice. The tumors were exposed to LIFU at the sound pressure of 0.053 MPa (Isppa: 91.2 mW cm^−2^) for 10 min every day during day 9 to day 22 after injection (Figures 6A, 6B). Twenty-two days post-transplantation, for the ultrasound treatment group, the tumor volume reached 197.51 ± 17 mm^3^ for A549 group, 72.30 ± 16 mm^3^ for the doxycycline-tet-on-V23A-MscL group, and 59.80 ± 8 mm^3^ for the I92GI96G-MscL group, and the tumor weights were 89.00 ± 9 mg, 50.00 ± 4 mg and 40.20 ± 6 mg, respectively (Figures 6C, D). Both activations of V23A-MscL and I92GI96G-MscL channels significantly suppressed tumor growth. Further comparison between no-US and US group showed a significant difference between I92GI96G group and I92GI96G-US group, whereas LIFU has no obvious effect on V23A-MscL expressing group (V23A-Dox vs. V23A-Dox-US, Figure 6E). H&E of tissue blocks further showed that tumor growth was partly suppressed by the activation of V23A-MscL and LIFU *via* I92GI96G-MscL, presented by the enlarged area of cell damage or death (**upper layer,** Figure 6F). TUNEL staining showed no significant difference in all groups here, demonstrating the absence of apoptosis in cell death induced by V23A-MscL-expressing or ultrasound-treated I92GI96G A549 tumors (**lower layer,** Figure 6F). These results demonstrated that the expression of V23A-MscL or ultrasound-induced activation of I92GI96G-MscL can suppress A549 tumor growth, suggesting potential anticancer activities of MscL and ultrasound. Confocal imaging of slice from tumors showed that stronger mCherry was detected in V23A-Dox group and V23A-Dox-US group, and the latter appeared more powerful (Supplementary Figure S3A). Whereas, the relationship of channel expression level and the observed effects remains unclear and needs further investigation.

## 4 Discussion

Due to the definitive mechanical gating and nanopore upon opening, there has been much interest in applying ultrasound to regulate mammalian cell function *via* MscL (Qiu et al., 2020). In this study, we further remodeled MscL to achieve a double-mutant I92GI96G that can respond to LIFU. We then found the important role of permeability of MIM in cytoplasmic vacuolization, demonstrating the successive morphological changes from cell swelling and cytoplasmic vacuolization to membrane blebbing. In addition, we revealed that LIFU induced opening of MscL can suppress the growth of NSCLC A549 tumors in a mouse model, suggesting the combination of MscL and ultrasound in cancer therapy.

When MscL was expressed in A549 cells, the observed effects have more relation to the opening degree of MscL than the expressing level, such as opening possibility, pore size and duration time. For GOF mutants like V23A and I92GI96G, the expression level is the primary cause of observed effects, and pore size difference causes the diverse effects between V23A and I92GI96G. MTSET induces locked opening of G26C-MscL channels and the cells shows blebbing and loss viability quickly. In addition, as long as the existence of MTSET, the cells with weak G26C-mCherry fluorescence (suggesting the minor expression of MscL) will bleb and stop move eventually, and the duration time is the leading cause. In our study, transfection, stable and inducible expression system were applied. We first used transient expression to study the influence of different subcellular located MscL on A549 cells. Compared to the expression of control vector (organelle-mTFP1), both V23A and I92GI96G cause evident cytoplasmic vacuolization. To further realize the application of MscL channel in animal model, lentivirus system was considered to construct stable expression. Although cytoplasmic vacuolization can be caused by overexpression of V23A and I29GI96G, stable cell line of I92GI96G can be acquired through antibiotic screening. However, normal stable cell line of V23A could not be acquired in the same setting. Herein, the mCherry fluorescence of I92GI96G indicating the expression of MscL is weaker than WT-MscL (Supplementary Figure S3B). That is, the degree of activation basing on expression level will influence the effect of MscL on cells.

On the other hand, cell death is a big and complex program (Galluzzi et al., 2018). Accumulating types of cell death were proved to be associated with pore forming proteins, and they may become useful regulator of cell death (Flores-Romero et al., 2020). Recently, two famous pore forming proteins MLKL and GSDMD have been demonstrated to have relation with necroptosis and pyroptosis, respectively (Shi et al., 2015; Samson et al., 2020). Drugs targeting GSDMs to treat cancer is on the design progress by Feng Shao team (PYROTECH, China). Compared MscL with MLKL or GSDMD, it has more simple mechanism and specific activation, making it easy for non-invasive cancer treatment by ultrasound. Cytoplasmic vacuolization is a morphological status during cell stress. Subsequent cell death after cytoplasmic vacuolization is accompanied by cell contraction and membrane blebbing, which are the characteristics of apoptosis and necrosis, respectively (Vanden Berghe et al., 2010). Cytoplasmic vacuolization is a kind of cell death related to cell metabolism, which shows stronger potentials in treatment of cancer caused by metabolic disorders.

A variety of factors including insulin-like growth factor I receptor, small-molecule compounds, photodynamic therapy, and synthetic drugs, are responsible for cytoplasmic vacuolization (Sperandio et al., 2000; Wang et al., 2019b; Kessel, 2020). However, unlike apoptosis, pyroptosis, and necroptosis that have relatively explicit signaling pathways, no uniform conclusion was achieved in the mechanism of cytoplasmic vacuolization associated cell death (Samson et al., 2020; Kessel, 2021). Paraptosis is the only type with cytoplasmic vacuolization that has been defined. Previous studies showed that ER and proteasome function are involved in cytoplasmic vacuolization (Chen et al., 2008). Herein, MIM-targeted MscL induced cytoplasmic vacuolization involved cell shrinkage, membrane blebbing, disruption of membrane permeability, deformed nuclei, and global membrane disorders. Permeability of mitochondria is believed to be essential in cell function (He et al., 2017; Zhou et al., 2019). Cytochrome-c can be released through GSDME-N pores formed in the mitochondrial membrane (Rogers et al., 2019). In addition, necrosis can be induced by the irreversible opening of the permeability transition pore complex (PTPC) (Izzo et al., 2016). However, no association between cytoplasmic vacuolization and mitochondrial permeability transition (MPT)-driven necrosis was found. In our study, cytoplasmic vacuolization was confirmed to be formed in MIM-targeted MscL-expressing cells, and we propose that the disruption of membrane permeability is a prime requirement for cytoplasmic vacuolization.

Mitochondria may have its own characteristics that suitable for MscL regulation. Previous studies confirm that MscL gates through force-from lipid principle and that it can be activated by any membrane curvature and/or transbilayer pressure profile independent of other proteins or ligands. The MIM is composed of the inner boundary membrane (IBM) and cristae, and the mitochondrial cristae remodeling includes widening and tightening of the cristae (Quintana-Cabrera et al., 2018). The morphological matrix changes involved in the local mechanical microenvironment of MIM may decrease the gating energy barrier and promote the MscL opening. Actually, WT-MscL and G26C-MscL were determined by higher gating threshold. However, partial cytoplasmic vacuolization was only seen in MITO-located WT-MscL- and G26C-MscL-expressing A549 cells, which was absent from other subcellular locations, indicating the more opening probability of both channels localized in MIM. Whether the changes in membrane tension formed by cristae remolding or proton gradient influence the gating of MscL channel remains for further analysis. Moreover, we confirmed the cytoplasmic vacuolization caused by increased permeability of the membrane in SK-HEP-1, MCF7, and MDA-MB-231 cells as well (data not shown). In addition, the increased permeability of the cell membrane may indicate potential efficient delivery of chemical drugs, which suggests us the correlation of drug delivery and lethality of MscL.

Using GOF-MscLs with different mechanosensitivities from mild to severe, we found that MIM-targeted MscL-expressing A549 cells underwent the progress from pre-vacuolization state with cellular swelling to cytoplasmic vacuolization, then ended with shrinkage and membrane blebbing that causes necrosis. The cell swelling may be caused by slight leakage of membrane from MscL pores and develop to cytoplasmic vacuolization due to more channel opening and more severe cell damage by sustained gating. These findings provide the underlying mechanism of cytoplasmic vacuolization and reveal the association between cytoplasmic vacuolization and necrosis, which can stimulate anticancer immune responses. MLKL forms a pore size much larger than MscL, however, no cytoplasmic vacuolization was seen in necroptosis. Cytoplasmic vacuolization has also not been observed in MPT-driven necrosis. Maybe the cytoplasmic vacuolization was ignored or just can’t be caught in these circumstances. Here, with various mechanosensitive GOF-MscLs, we have increased possibilities to observe the subtle modification of the cellular process. Considering the expression changes of caspase-8, caspase-9, and LC3A/B in cytoplasmic vacuolization induced by curcumin and MG132, apoptosis and autophagy were suggested to be involved. Further investigation should be addressed on the crosstalk of signaling pathways in different types of cell death.

Ultrasound is an excellent method to perform targeted stimulation due to its non-invasiveness and precise localization. Proteins with mechanosensitivity are believed to be an effective mechano-transducer in sonogenetics. In consideration the safety and sensitivity of the application, we are seeking for efficient ultrasound stimulation with sound pressure as low as possible *via* MscL. Herein, 0.044 MPa is sufficient to directly activate I92GI96G-MscL in A549 cells without the present of microbubbles. Previous study showed 0.3 MPa for activating neurons expressing G22S-MscL (Qiu et al., 2020). In addition, G22S-MscL mutant can be activated by sound pressure between 0.047 MPa and 0.095 MPa with microbubbles in retinal pigment epithelial cells (Heureaux et al., 2014). Moreover, compared with the sound pressure of 0.45 MPa for activating I92L-MscL in hippocampal neurons (Ye et al., 2018), 0.176 MPa or 0.6 MPa for activating PIEZO1 with microbubbles (Pan et al., 2018), the sound pressure of 0.053 MPa we used here proved to be effective in mice tumor suppression. A mammalian auditory protein prestin is also proved to be activated by ultrasound for modulating cellular functions at the sound pressure of 0.5 MPa (Huang et al., 2020; Wu et al., 2020). In contrast to PIEZO1 and prestin, MscL channels usually respond to lower intensity of ultrasound and have smaller molecule weight, suggesting its remarkable potential in sonogenetics. In our study, ultrasound treatment showed obvious inhibition in the initial days of tumor growth, however, the anticancer effect is limited upon increasing tumor size. Therefore, two questions remained unsolved. One is the available ultrasound device with bigger focus size, the other is a more suitable MscL modification with appropriate ultrasonic response. Along with the optimization and further application of MscL, the anticancer effect can be achieved through precise spatiotemporal control of MscL channels.

## 5 Conclusion

In summary, we found morphological anomalies developing with the degree of MscL opening and confirmed that the permeability of mitochondria inner membrane played an essential role in cytoplasmic vacuolization. The mechanism underlying cytoplasmic vacuolization has crosstalk with apoptosis and autophagy. Moreover, MscL can serve as a nanomedicine and a mechanical transducer. Low intensity focused ultrasound can suppress A549 tumor growth *via* activating MscL, providing the theoretical and experimental basis for the application of MscL and ultrasound in anticancer therapy.

## Data Availability

The original contributions presented in the study are included in the article/Supplementary Material, further inquiries can be directed to the corresponding authors.
