# Supplementary material for: Mechanosensitive channel MscL induces non-apoptotic cell death and its suppression of tumor growth by ultrasound
Source: Front Chem. 2023 Mar 1;11:1130563. doi: 10.3389/fchem.2023.1130563 (PMC10014542; doi:10.3389/fchem.2023.1130563)
Supplement: Supplementary file 1 [file Image2.pdf]

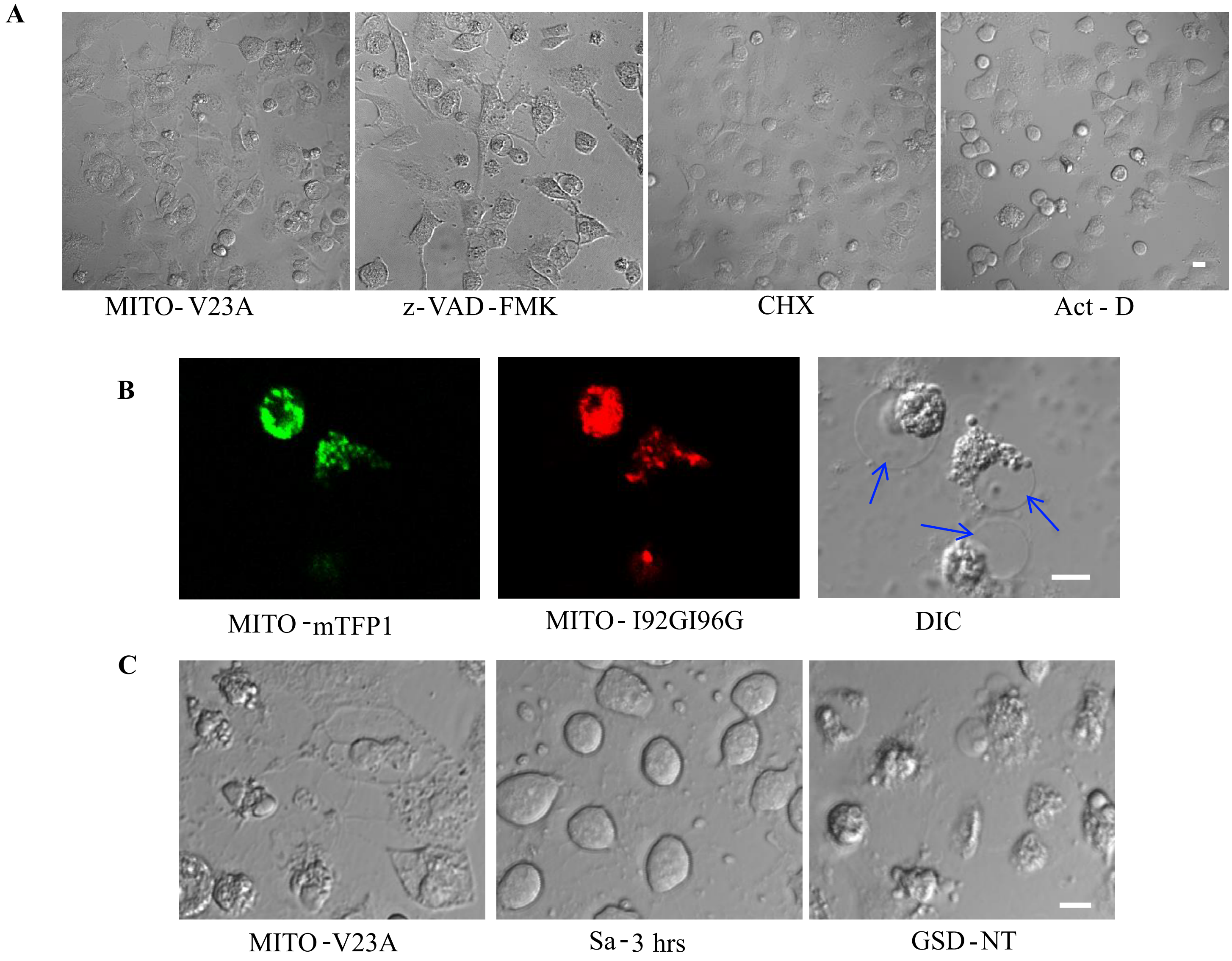

Figure S2. Spontaneous opening of MscL caused cytoplasmic vacuolization and its inhibition. (A) Representative confocal imaging of A549 cells in response to indicated inhibitors, z-VAD-FMK (25 µM, 12 h), Cycloheximide (CHX, 10 µg mL-1, 12 h), Actinomycin D (Act-D, 1 µg mL-1, 12 h). (B) Confocal imaging of transiently MITO-I92GI96G-MscL expressing A549 cells at 72 h after transfection. (C) Morphological comparison of A549 cells with cytoplasmic vacuolization (overexpression of MITO-V23A-MscL), undergoing apoptosis (starved overnight, Staurosporine (Sa) 1 µM, 3 h) and pyroptosis (Gasdermin D-N-terminal (GSD-NT), 24 h). Scale bars: 10 µm.
