# Supplementary material for: Mechanosensitive channel MscL induces non-apoptotic cell death and its suppression of tumor growth by ultrasound
Source: Front Chem. 2023 Mar 1;11:1130563. doi: 10.3389/fchem.2023.1130563 (PMC10014542; doi:10.3389/fchem.2023.1130563)
Supplement: Supplementary file 2 [file Image3.pdf]

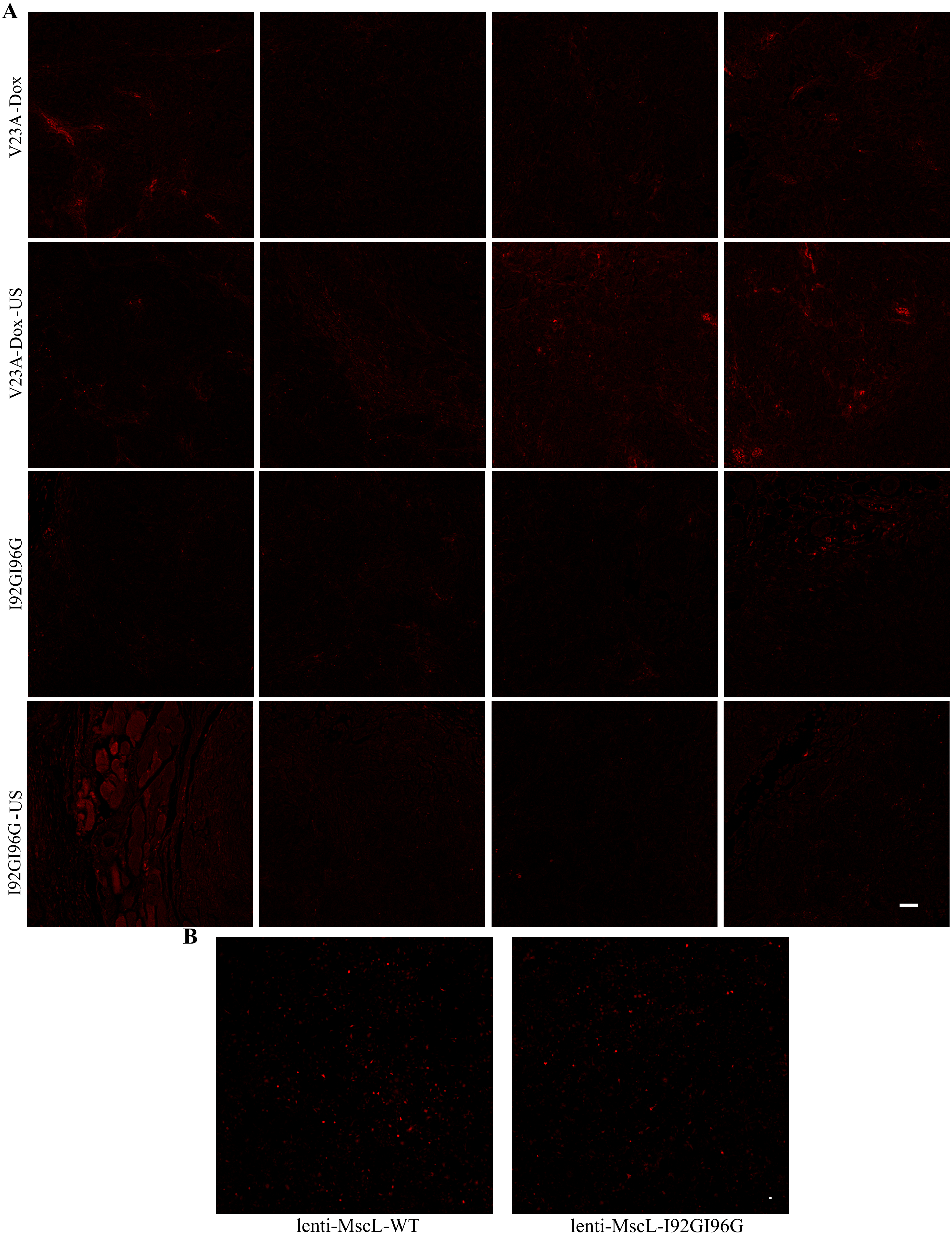

Figure S3. (A) Confocal imaging of MscL-mCherry in obtained tumors from mice, every image is from different mice. Scale bar: 50 μm. (B) MCherry indicates the expression of MscL in lentivirus stable A549 cells. Scale bars: 10 μm.
