# Supplementary material for: Mechanosensitive channel MscL induces non-apoptotic cell death and its suppression of tumor growth by ultrasound
Source: Front Chem. 2023 Mar 1;11:1130563. doi: 10.3389/fchem.2023.1130563 (PMC10014542; doi:10.3389/fchem.2023.1130563)
Supplement: Supplementary file 4 [file Image1.pdf]

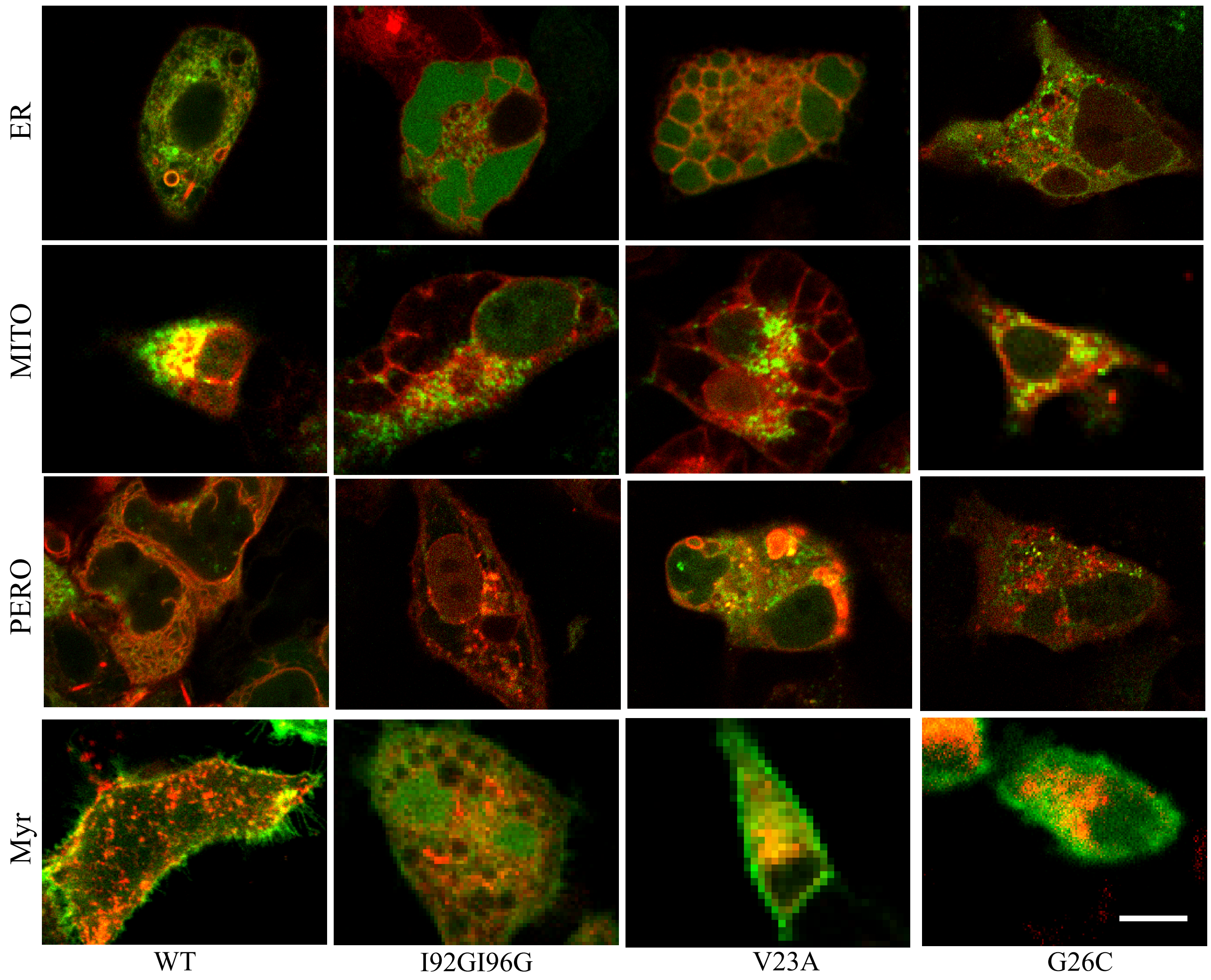

Figure S1. Confocal imaging of co-localization of organelle-mTFP1 and different subcellular-MscL in A549, Scale bars: 10 μm.
